# Exosome-Based Multivalent Vaccine: Achieving Potent Immunization, Broadened Reactivity, and Strong T-Cell Responses with Nanograms of Proteins

**DOI:** 10.1128/spectrum.00503-23

**Published:** 2023-04-24

**Authors:** Mafalda Cacciottolo, Justin B Nice, Yujia Li, Michael J. LeClaire, Ryan Twaddle, Ciana L. Mora, Stephanie Y. Adachi, Esther R. Chin, Meredith Young, Jenna Angeles, Kristi Elliott, Minghao Sun

**Affiliations:** a Capricor Therapeutics, Inc., San Diego, California, USA; National Institutes of Health

**Keywords:** exosome, severe acute respiratory syndrome coronavirus 2, spike, nucleocapsid, neutralizing antibodies, omicron, lentiviral system, COVID, vaccine, therapeutic

## Abstract

Currently approved vaccines against severe acute respiratory syndrome coronavirus 2 (SARS-CoV-2) have focused solely on the spike protein to provide immunity. The first vaccines were developed rapidly using spike mRNA delivered by lipid nanoparticles but required ultralow-temperature storage and have had limited immunity against variations in spike. Subsequently, protein-based vaccines were developed, which offer broader immunity but require significant time for development and the use of an adjuvant to boost the immune response. Here, exosomes were used to deliver a bivalent protein-based vaccine in which two independent viral proteins were used. Exosomes were engineered to express either SARS-CoV-2 delta spike (Stealth X-Spike [STX-S]) or the more conserved nucleocapsid (Stealth X-Nucleocapsid [STX-N]) protein on the surface. When administered as a single product (STX-S or STX-N) or in combination (STX-S+N), both STX-S and STX-N induced strong immunization with the production of potent humoral and cellular immune responses. Interestingly, these results were obtained with the administration of only nanograms of protein and without an adjuvant. In two independent animal models (mouse and rabbit), the administration of nanograms of the STX-S+N vaccine resulted in increased antibody production, potent neutralizing antibodies with cross-reactivity to other variants of spike, and strong T-cell responses. Importantly, no competition of immune responses was observed, allowing the delivery of nucleocapsid with spike to offer improved SARS-CoV-2 immunity. These data show that the StealthX exosome platform has the enormous potential to revolutionize vaccinology by combining the advantages of mRNA and recombinant protein vaccines into a superior, rapidly generated, low-dose vaccine resulting in potent, broader immunity.

**IMPORTANCE** The pandemic emergency has brought to light the need for a new generation of rapidly developed vaccines that induce longer-lasting, potent, and broader immune responses. While the mRNA vaccines played a critical role during the emergency in reducing SARS-CoV-2 hospitalization rates and deaths, more efficient approaches are needed. A multivalent, protein-based vaccine delivered by exosomes could meet this urgent need due to the high speed of development, manufacturability, and the ability to produce a strong antibody response, with neutralizing antibodies and a strong T-cell response able to broadly combat viral infection with a minimum number of injections.

## INTRODUCTION

The severe acute respiratory syndrome coronavirus 2 (SARS-CoV-2) pandemic has brought to light the need for better vaccines that can be rapidly generated to protect the population broadly against viruses and their consequent morbidity. More recently, increasing numbers of cases of SARS-CoV-2 caused by constantly changing variants of spike, together with the surge of influenza virus and respiratory syncytial virus (RSV), have demonstrated the urgent need for multivalent vaccines that could improve SARS-CoV-2 immunity while also preventing infection by other viruses.

Exosomes offer a new antigen delivery system that could be utilized to rapidly generate multivalent protein-based vaccines. Exosomes, first identified as extracellular membrane vesicles (1, 2), are small vesicles of ~30 to 200 nm in diameter that are enriched in specific subsets of proteins (CD81, CD63, and CD9 [tetraspanin family]), RNAs, and lipids (3) and responsible for cell-to-cell communication. Being an endogenous cell product, exosomes have extremely low immunogenicity and toxicity. Purification and scale-up processes for exosomes rely on well-established particle protocols utilizing bioreactors and differential filtration for the manufacturing process. Thus, exosomes can be rapidly engineered to express targets of interest and easily produced for use in multivalent vaccines.

SARS-CoV-2 has been the promoter of scientific advancement in the vaccinology field, resulting in the rapid development and approval of new mRNA-based vaccines from Moderna and Pfizer (4, 5) and the subsequent approval of protein-based vaccines from Novavax (6, 7). These vaccines were developed to direct the immune response to the surface spike (S) protein, which binds to the host cell receptor angiotensin-converting enzyme 2 (ACE2), mediating viral cell entry and infection (8). The S protein, a class I fusion glycoprotein and a major surface protein on SARS-CoV-2, mediates binding to the ACE receptor on cell surfaces, promoting its propagation and infection, and is the primary target of neutralizing antibodies (9). Since the beginning of the coronavirus disease 2019 (COVID-19) pandemic, SARS-CoV-2 has mutated extensively, reducing the efficacy of current vaccines, particularly mRNA-based vaccines, and has created the need for more effective approaches to immunization (10, 11).

The SARS-CoV-2 genome encodes three additional structural proteins that are important for the biology of the virus: nucleocapsid (N or Ncap), envelope, and membrane. The nucleocapsid protein, in contrast to spike, is highly conserved, with 90% amino acid homology and fewer mutations over time (12). Ncap binds to the mRNA during the packaging of the RNA genome, regulates the synthesis of viral RNA during replication and transcription, and modulates its metabolism in infected subjects. IgG antibodies against Ncap have been detected in SARS patients (13, 14), with significant prognostic value (14). Despite not being a target of neutralizing antibodies, the Ncap protein can stimulate the T-cell response (15) and improve the efficacy of next-generation vaccines (16).

While the mRNA-based vaccines were of undeniable help during the initial response to the SARS-CoV-2 pandemic, they lack long-term protection (17), likely due to unknown translation efficiencies and the lack of cross-reactivity with new variants of concern (VOCs). Thus, there is a pressing demand to develop next-generation COVID-19 vaccine strategies (18). While many approaches could be taken into consideration, updating the spike antigen to match the circulating VOCs seems to be the easiest option; however, this may result in the inaccurate identification of the correct variant and therefore may impact vaccine efficacy, as has been observed for influenza virus and, more recently, SARS-CoV-2 (19). An alternative approach could be a “mix-and-match” approach in which the spike antigen is formulated together with a more conserved viral antigen such as nucleocapsid for SARS-CoV-2. This approach would broaden the immune response at both the humoral and T-cell levels and result in more efficient protection against current and future forms of the virus.

We have shown that immunization with our exosome-based SARS-CoV-2 delta spike vaccine was able to induce a potent, broad immune response (see Fig. 2). These results were achieved with only nanograms of spike protein delivered by exosomes without any adjuvants (see Fig. 2). Therefore, by utilizing our exosome platform (StealthX [STX]) to engineer exosomes to express the SARS-CoV-2 delta spike protein (STX-S) or nucleocapsid protein (STX-N) on their surface, we were able to produce a cocktail of exosomes to create a bivalent vaccine against SARS-CoV-2 named STX-S+N. The administration of the STX-S+N vaccine in mice and rabbits resulted in increases in both CD4^+^ and CD8^+^ T-cell responses, accompanied by a potent humoral immune response, as demonstrated by high levels of neutralizing antibodies against not only delta SARS-CoV-2 but also the omicron variants. Importantly, these results were observed after STX vaccination using only nanogram concentrations of SARS-CoV-2 proteins presented by exosomes in the absence of any adjuvant or synthetic lipid nanoparticles, which further strengthens the STX exosome safety profile as a vaccine candidate. Additionally, due to the significantly smaller concentration of protein (nanograms for STX versus milligrams for standard recombinant protein vaccines) required to induce a robust immune response, this study opens the door to multivalent vaccines using multiple SARS-CoV-2 antigens in combination with influenza virus and/or RSV for a much broader and safer future immunization campaign.

These results support the clinical development of the STX vaccine for immunization against SARS-CoV-2.

## RESULTS

### SARS-CoV-2 protein expression on the surface of STX-producing cells and exosomes.

**FIG 1:**
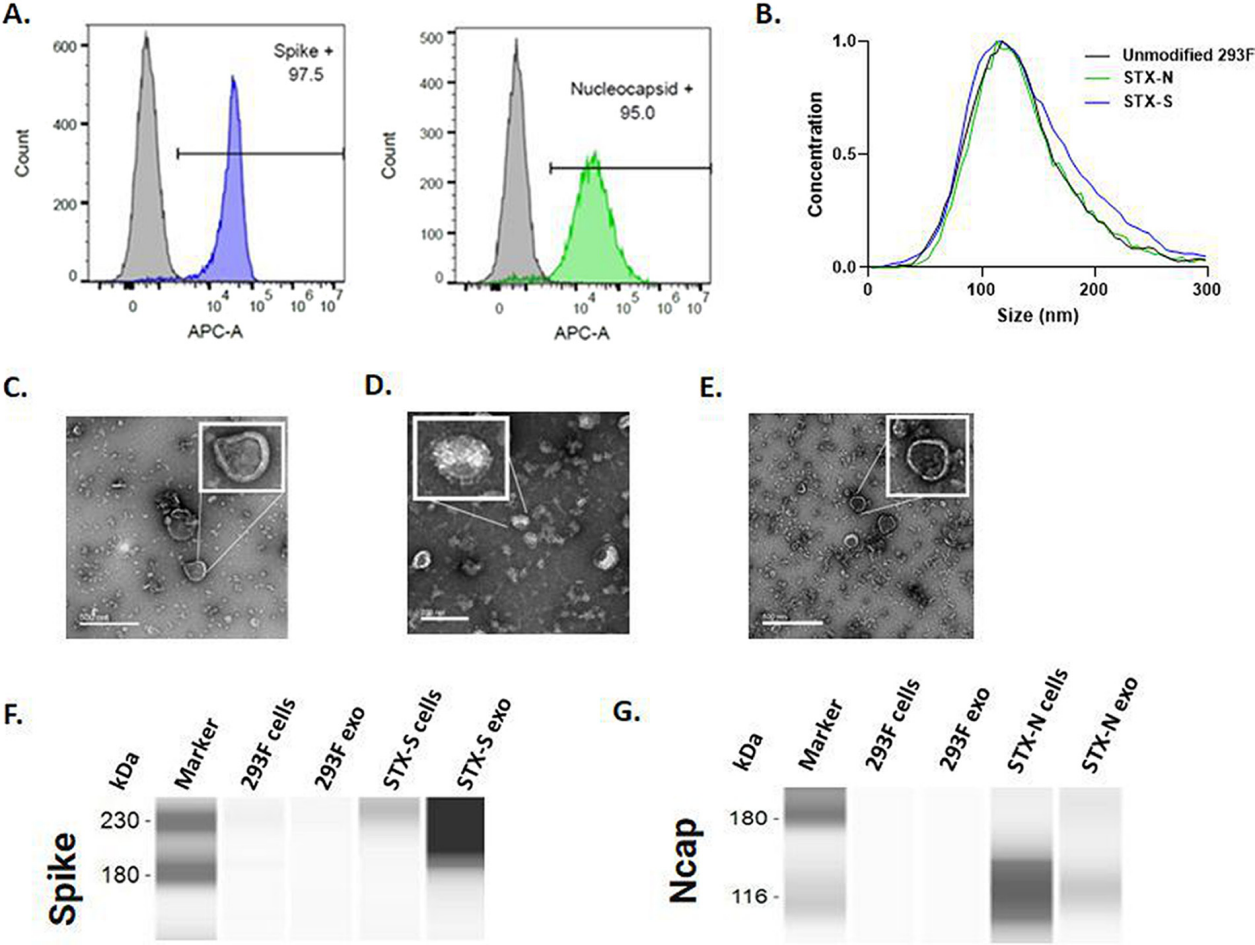
Characterization of SARS-CoV-2 proteins on cell and exosome surfaces. (A) Representative images of the gating strategy for the analysis of protein expression on the cell membrane. High levels of expression of spike (blue histogram) and nucleocapsid (green histogram) were detected on the cell surface by flow cytometry. Parent, nonengineered 293F cells do not express SARS-CoV-2 antigens, as expected (gray histograms). (B) Size distributions of STX-S and STX-N exosomes by ZetaView nanoparticle tracking analysis (NTA). (C to E) TEM images of purified naive 293F cells (C) and STX-S (D) and STX-N (E) exosomes (exo). (F and G) Spike (F) and nucleocapsid (G) expression analyzed by Jess automated Western blotting. A total of 0.8 μg of protein was loaded per lane, as calculated by a BCA assay.

STX cells were generated by lentiviral transduction, and the expression of SARS-CoV-2 proteins on the cell surface was evaluated by flow cytometry (Fig. 1). As shown, STX cells showed >95% increased expression of spike and nucleocapsid (Fig. 1A) compared to that in parental 293F cells.

STX exosomes were purified from the engineered 293F cell culture supernatant using Capricor’s laboratory-scale purification technique (see Materials and Methods).

The purified STX-S and STX-N exosomes showed expected average diameters of 144.8 nm and 140.1 nm (Fig. 1B; see also Table S1 in the supplemental material) and an expected polydispersity index (PDI) of <0.2 (0.152 and 0.129) (Fig. 1B and Table S1), respectively (20).

STX exosomes were analyzed by transmission electron microscopy (TEM) imaging. As shown in Fig. 1C to E, typical exosome sizes and morphologies were observed, with the detection of round, smooth nanoparticles that had a visible lipid bilayer. Importantly, spike protrusions were visible on the surface of the STX-S nanoparticles, indicating the presence of spike protein on exosomes (Fig. 1D). For nucleocapsid, a characteristic lipid bilayer was observed, which resulted in thicker-than-naive exosomes (Fig. 1C), suggesting an accumulation of particles in the exosome membrane (Fig. 1E).

Spike and nucleocapsid expressions were verified in cell lysates and exosomes using Protein Simple’s Jess automated Western blot assay. Spike protein was detected in both STX-S cells and exosomes, with an enrichment of spike protein in the exosome samples. Engineered STX-N cells and exosomes expressed abundant nucleocapsid (Fig. 1F and G): while trafficking to the exosome is confirmed, the efficacy of trafficking might be lower for soluble proteins (i.e., Ncap) (Fig. 1G) than for transmembrane proteins (i.e., spike) (Fig. 1F). Furthermore, the SARS-CoV-2 proteins spike and Ncap were detected on the exosome membrane using a bead-based CD81 assay, with >75% expression together with the exosome-specific marker CD81.

The concentrations of spike antigen in STX-S exosomes and nucleocapsid antigen in STX-N exosomes were further quantified by an enzyme-linked immunosorbent assay (ELISA). A final STX-S preparation of 1 × 10^12^ exosomes/mL contains on average 253.77 ng of spike, while a final STX-N preparation of 1 × 10^12^ exosomes/mL contains on average 40.59 ng of nucleocapsid.

### STX-S and STN-N individually induced strong immunization in the absence of an adjuvant.

**FIG 2:**
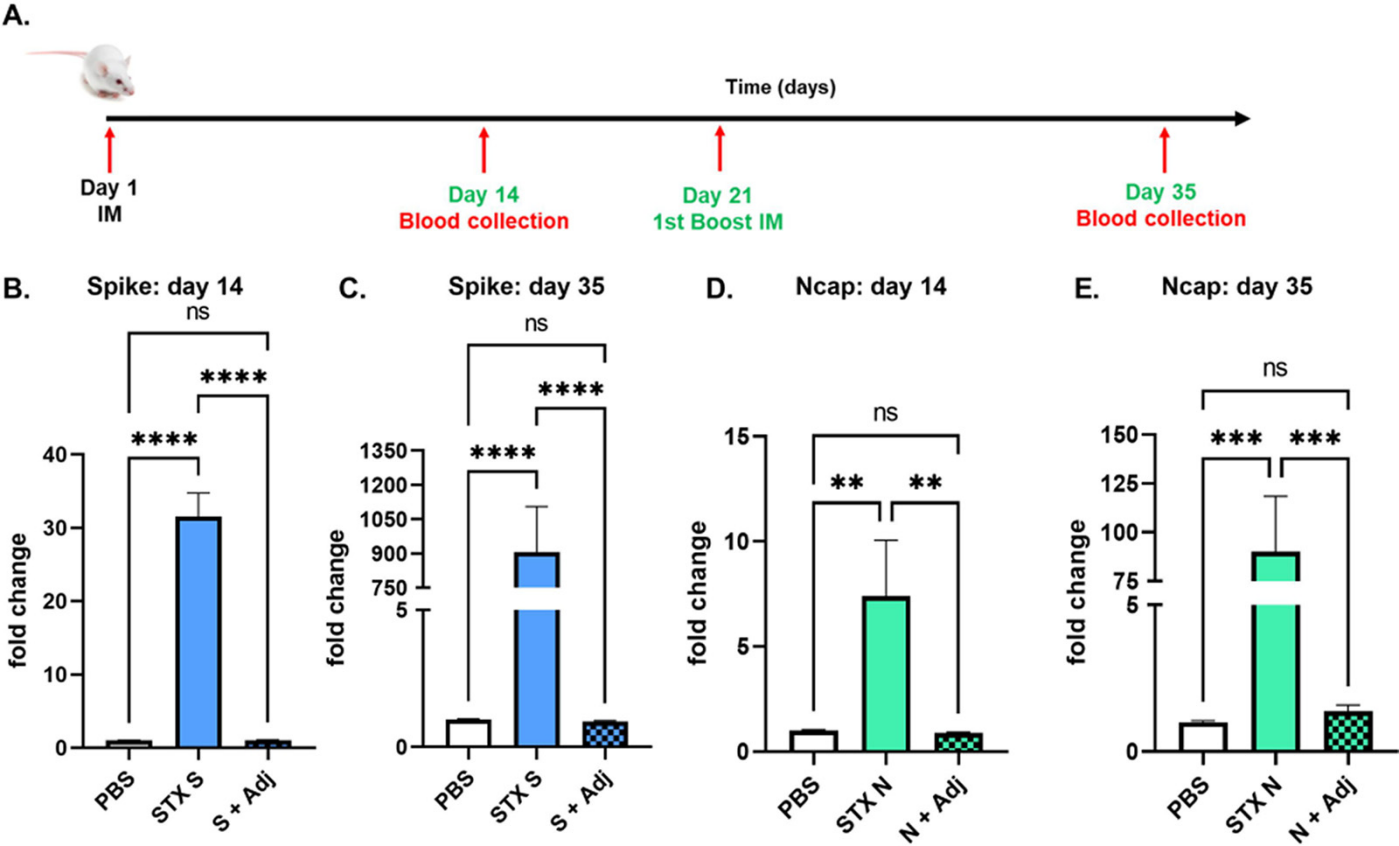
STX-S and STX-N exosomes induce strong immunization without an adjuvant. (A) Timeline of the study. (B) Increased IgG production against spike after 1 i.m. injection. (C) Increased IgG production against spike after 2 i.m. injections. (D) Increased IgG production against Ncap after 1 i.m. injection. (E) Increased IgG production against Ncap after 2 i.m. injections. Spike (S + Adj) and Ncap (N + Adj) proteins at the same doses in combination with an adjuvant are shown. Data are shown as means ± SEM (*n* = 10 per experimental group). ****, *P* < 0.0005; ***, *P* < 0.001; **, *P* < 0.01; ns, not significant (by 1-way ANOVA).

To validate the ability of STX-S and STX-N exosomes to induce an immune response, mice were immunized with a 10 ng exosome formulation of STX-S and STX-N. As a comparison to show the robustness of exosome delivery, 10 ng of either spike or Ncap recombinant protein was delivered in combination with the adjuvant (Alhydrogel; InvivoGen). Phosphate-buffered saline (PBS) was used as a negative control. Blood collected 2 weeks after the boost injection (2nd injection) showed that both the STX-S and STX-N vaccines increased antibody production against spike and nucleocapsid, respectively, in all animals. Neither the spike nor the Ncap protein in combination with the adjuvant was statistically different from the PBS control, and no antibody production was observed (Fig. 2). 293F exosomes were not included as a control since the immune response at both the antibody and interferon gamma (IFN-γ) production levels was comparable to that with PBS (Fig. S1).

### The STX-S+N vaccine induces strong immunization against SARS-CoV-2 protein in mice.

We proceeded to assess the immune response of a multivalent vaccine obtained by the combination of STX-S and STX-N exosomes. STX-S+N was administered to mice at three different doses (Table 1) by two intramuscular (i.m.) injections. A second i.m. injection, a boost injection, was delivered after a 3-week interval. PBS was used as a negative control in the study.

**TABLE 1:** Concentrations of spike and Ncap used in the STX-S+N mouse study

Dose	Route of injection^*a*^	Vol (μL)	Spike concn (ng/injection)	Ncap concn (ng/injection)
1	i.m.	100	25	2.5
2	i.m.	100	10	4
3	i.m.	100	3	9

a i.m., intramuscular.

**FIG 3:**
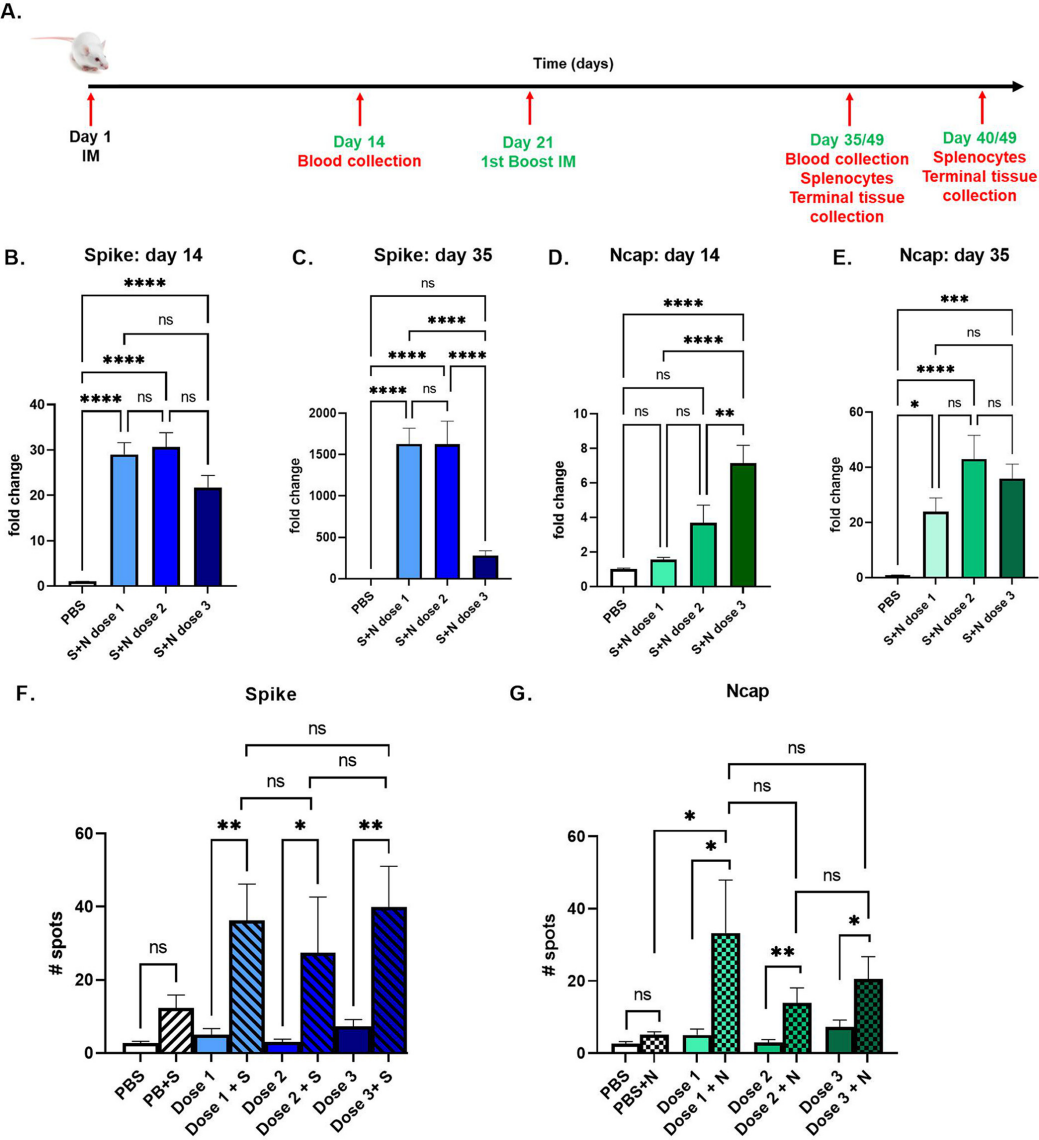
The STX-S+N vaccine elicits strong antibody and cellular responses in mice. The STX-S+N vaccine induced increases in antibodies against SARS-CoV-2 spike and nucleocapsid and a strong T-cell response. (A) Timeline of the mouse study. (B) IgG against spike on day 14. (C) IgG against spike on day 35. (D) IgG against Ncap on day 14. (E) IgG against Ncap on day 35. PBS was used as a vehicle control. (F) The IFN-γ response is increased after spike stimulation. (G) The IFN-γ response is increased after Ncap stimulation. Dose + Spike, splenocyte isolated from the experimental group and stimulated *in vitro* with spike protein; Dose + Ncap, splenocytes isolated from the experimental group and stimulated *in vitro* with Ncap protein. Data are shown as means ± SEM. *, *P* < 0.05; **, *P* < 0.01; ***, *P* < 0.005; ****, *P* < 0.001; ns, not significant (by 1-way ANOVA with adjustment for multiple comparisons) (*n* = 10 animals per experimental group).

Immunization induced by the STX-S+N vaccine was evaluated by the quantification of antibodies against Ncap and spike (Fig. 3B to E). An increase in antibody production was detected after the first injection and continued to increase after the boost injection (2nd injection). A single injection of STX-S+N induced an increase of up to 30-fold in IgG against spike, with no significant difference overall among the three doses. After complete immunization, dose 1 (25 ng spike) and dose 2 (10 ng spike) resulted in a 1,500-fold increase in antibody against spike, while dose 3 (3 ng spike [a significantly smaller amount of spike]) resulted in a 280-fold increase. On the other hand, a dose response was observed for Ncap. An increase of 1.5-fold was observed for low dose 1 (2.5 ng Ncap), an ~4-fold increase was observed for dose 2 (4 ng Ncap), and up to a 7-fold increase was observed for dose 3 (9 ng Ncap). After the complete immunization cycle (2 i.m. injections), no significant difference was observed across doses, with an increase in IgG against Ncap of between 24- and 43-fold being observed in STX-S+N-treated mice.

To characterize the T-cell response to STX-S+N, antigen-specific T-cell responses were measured by an enzyme-linked immunosorbent spot (ELISpot) assay (Fig. 3F and G). Vaccination with STX-S+N elicited multifunctional, antigen-specific T-cell responses. Splenocytes were isolated from animals on day 35 (2 weeks after the boost [2nd] injection) and evaluated using ELISpot plates precoated with IFN-γ. PBS was used as a control in the study, as described above. The baseline expression level was compared to that with stimulation with 10 μg/mL of either the spike or Ncap protein (Acro Biosystems). While baseline IFN-γ responses were comparable between groups, evaluation of IFN-γ-secreting cells in response *ex vivo* to either spike (Fig. 3F) or Ncap (Fig. 3G) stimulation showed a strong increase in spleens immunized with the STX-S+N vaccine, suggesting a Th1-biased CD8^+^ T-cell response. After spike stimulation, an average of a 7-fold increase in the IFN-γ response was observed despite the STX-S+N dose administered (Fig. 3F). After Ncap stimulation, a dose-response effect was observed, with a 7-fold increase in mice receiving the lowest Ncap dose (dose 1) (2.5 ng) and an ~3-fold increase in mice receiving either dose 2 (4 ng Ncap) or dose 3 (9 ng Ncap) (Fig. 3G).

### The STX-S+N vaccine induces strong immunization against SARS-CoV-2 protein in rabbits.

The immune response to the STX-S+N vaccine using a clinically relevant dose was evaluated in rabbits. The STX-S+N vaccine was administered to rabbits at two different doses (Table 2) by two i.m. injections. A second i.m. injection, a boost injection, was delivered after a 2-week interval. PBS was used as a negative control in the study.

**TABLE 2:** Concentrations of spike and Ncap used in the STX-S+N rabbit study^*a*^

Dose	Route of injection^*a*^	Vol (μL)	Spike concn (ng/injection)	Ncap concn (ng/injection)
1	i.m.	500	125	10
2	i.m.	500	50	20

a i.m., intramuscular.

**FIG 4:**
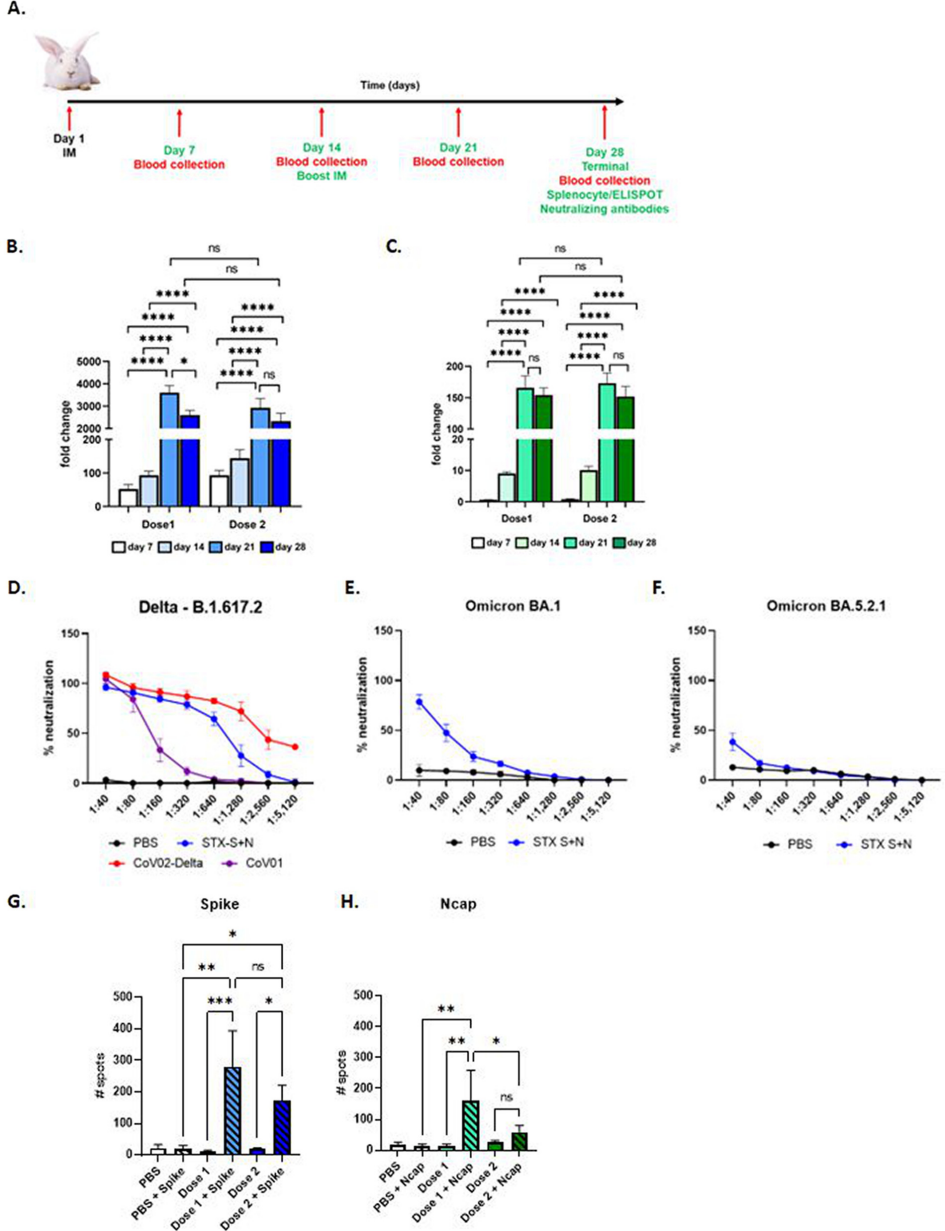
The STX-S+N vaccine elicits strong antibody and T-cell responses in rabbits. The STX-S+N vaccine induced statistically significant increases in antibodies against SARS-CoV-2 spike (B) and nucleocapsid (C). PBS was used as a vehicle control. Data are reported as fold changes over PBS (*n* = 8 for STX-S+N per dose, and *n* = 8 for PBS). (A) Timeline of the rabbit study. (B) IgG against spike. (C) IgG against Ncap. (D to F) Neutralizing antibodies. (D) The STX-S+N vaccine generated strong neutralization against SARS-CoV-2 delta spike (B.1.617.2). (E) The STX-S+N vaccine resulted in the neutralization of SARS-CoV-2 omicron BA.1 spike. (F) The STX-S+N vaccine resulted in the neutralization of SARS-CoV-2 omicron BA.5.2.1 spike. (G and H) T-cell response. (G) IFN-γ response after spike stimulation. (H) IFN-γ response after Ncap stimulation. COV-02-Delta, plasma from a patient immunized with Moderna’s mRNA vaccine with a breakthrough SARS-CoV-2 delta spike infection; CoV-01, plasma from a patient who received two doses of the Moderna COVID-19 vaccine and had no history of SARS-CoV-2 infection. Data are shown as means ± SEM. *, *P* < 0.05; **, *P* < 0.01; ***, *P* < 0.005; ns, not significant (by 1-way ANOVA with adjustment for multiple comparisons) (*n* = 8 animals per experimental group).

Increased antibody production was detected as early as 1 week after the first injection, which continued to increase after the boost injection (Fig. 4). After the complete immunization cycle (two i.m. injections), up to a 3,600-fold increase in IgG against spike (Fig. 4B) and up to a 170-fold increase in IgG against Ncap (Fig. 4C) were observed in STX-S+N-treated rabbits. No significant differences were observed between doses in antibody production against either antigen.

Immunization elicited by the STX-S+N vaccine was further evaluated by assessing neutralizing antibodies against SARS-CoV-2 variants (Fig. 4D to F). Plasma from rabbits given dose 2 (50 ng S and 20 ng N [8 animals]) and 2 PBS controls were tested for neutralizing antibodies against the SARS-CoV-2 delta variant. Potent neutralizing activity was elicited by STX-S+N in all analyzed animals. Importantly, STX-S+N in rabbits induced a response comparable to that in human control plasma (COV-02-delta [plasma from a patient fully immunized with Moderna’s mRNA vaccine with breakthrough delta infection]), with a complete neutralizing response at higher dilutions (i.e., 1:320 [range of 116.74 to 881.47% and average of 96.22% ± 8.9%]) (Fig. 4D). Moreover, STX-S+N in rabbits performed better than the CoV-01 control (plasma from a patient who received two doses of the Moderna COVID-19 vaccine and had no history of SARS-CoV-2 infection).

Additionally, the same samples were tested for neutralizing antibodies against SARS-CoV-2 omicron variants (omicron BA.1 and BA.5.2.1). As shown in Fig. 4E and F, strong cross-neutralization was observed for the STX-S+N-treated rabbits, achieving an average neutralization of 75% (range of 40.7 to 100%) for omicron BA.1 (Fig. 4E) and a range of between 10% and 91% for omicron BA5 (Fig. 4F). These data suggest that protein-based vaccines delivered by exosomes, specifically STX-S+N, may result in broader protection against SARS-CoV-2 variants. In all assays, rabbits receiving PBS showed no neutralization.

Finally, the T-cell response to STX-S+N immunization was measured by an ELISpot assay (Fig. 4G and H). Vaccination with STX-S+N elicited multifunctional, antigen-specific T-cell responses. Splenocytes were isolated from animals on day 28 (2 weeks after the boost [2nd] injection) and evaluated using ELISpot plates precoated with IFN-γ. PBS was used as the control in the study, as described above. The baseline expression level was compared to that with stimulation with 10 μg/mL of either the spike or Ncap protein (Acro Biosystems). While the baseline IFN-γ responses were comparable between groups, stimulation with either spike (Fig. 4G) or Ncap (Fig. 4H) resulted in a strong increase in IFN-γ production in spleens immunized with the STX-S+N vaccine, suggesting a Th1-biased CD8^+^ T-cell response. For spike, a clear dose response was observed, with a greater response in rabbits receiving dose 1 (125 ng S) (+28-fold) than in those receiving dose 2 (50 ng S) (+10-fold), although the increased response was not statistically significant (Fig. 4G). For Ncap, dose 1 (10 ng N) (+10-fold) resulted in a better immune response than dose 2 (20 ng N) (+2-fold) (Fig. 4H).

## DISCUSSION

The SARS-CoV-2 pandemic has brought to light the need for a new generation of rapidly developed vaccines that induce longer-lasting, potent, and broader immune responses to combat the ever-changing variants of SARS-CoV-2 and hinder virus transmission. While the mRNA vaccines played a critical role during the initial response to the pandemic in reducing SARS-CoV-2 hospitalization rates and deaths, its short-lived immunity and reduced ability to protect against new VOCs have pushed the scientific community to look for new approaches.

A multivalent, protein-based vaccine delivered by exosomes could meet this urgent need for a new generation of vaccines due to the high speed of development, manufacturability, and the ability to produce a strong antibody response, with neutralizing antibodies and a strong T-cell response able to broadly combat viral infection with a minimum number of injections.

Here, we have shown that exosomes can be used to deliver viral proteins for immunization. Our StealthX (STX) platform generated two vaccine candidates (STX-S and STX-N) that independently, and in combination (STX-S+N), induced a strong immune response against two SARS-CoV-2 proteins when combined in a single shot in two different animal models by delivering nanograms of proteins on the surface of the exosomes. No adjuvant was needed, <100-fold less protein was used than in traditional recombinant protein vaccines (21), and no competition between proteins was observed.

The “multivalent” or “combination” vaccines have multiple advantages from a public health standpoint (22). They require fewer shots, which would positively engage the population, increasing the percentage of vaccinated people, with broader epidemiological benefits. Consequently, fewer shots due to combination vaccines also decrease the overall cost of vaccinating a large population. Of course, there are limitations that need to be taken into consideration. First, it is crucial that the selected antigens contain sequences as highly conserved as possible, with low mutagenesis rates, in combination with the most recent, prevalent variants of concern. Importantly, immunogen interference is critical: minimal to no competition should be observed when using a multivalent vaccine. As demonstrated by STX-S+N, the multivalent vaccine shows the same strength of efficacy as the single product, with thousandfold increases in antibody loads postvaccination. Additionally, STX-S+N elicited both quantitative and qualitative immune responses (23): a consistent increase in the amount of antibody produced, the presence of protection as delineated by neutralizing antibodies, and the engagement of T cells were all observed in response to the administration of our exosome vaccine STX-S+N. Importantly, no deleterious side effects were recorded: both mice and rabbits showed no changes in weight or blood tests and no alteration at the tissue level (see Fig. S2 and Table S3 in the supplemental material), suggesting an overall good safety profile. STX-S+N has an advantage over the single-antigen vaccine. Conceptually, most of the immune protection against the virus is due to the antibody response to surface proteins (i.e., spike), the main targets of neutralizing antibodies. Immunization with a more conserved viral protein, such as nucleocapsid, adds an additional line of defense, stimulating T cells, which is especially important in the case of a highly mutating virus that escapes immune system defenses and is responsible for recurring breakthrough infections.

Our data suggest that exosomes are ideal vehicles for vaccination because they can safely deliver the antigen of interest (exogenous protein) efficiently by mimicking natural viral infection. Exosome-based vaccines constitute an innovative approach for an efficient virus-free, human-derived vaccine design (23). Yoo et al. observed that exosomes or extracellular vesicles at large could support the vaccination needs beyond traditional strategies: compared to viral or vector methods, exosomes are not immunogenic themselves but are carriers of a protein that retains the original conformation, tridimensional structure, and modifications, all embedded in its lipid bilayer membrane and ready to be efficiently presented as such to the immune system (23).

Importantly, the strong T-cell response initiated by STX-S+N vaccine administration is clinically relevant and crucial for the new generation of COVID vaccines. Keeton et al. observed that while neutralizing antibodies might not recognize new VOCs, the T-cell population is able to cross-react with them and confer protection (24). We have observed robust spike- and Ncap-specific T-cell responses: the highly conserved nucleocapsid protein used in the STX-S+N vaccine could further increase and broaden the efficacy of SARS-CoV-2 vaccines, engaging an additional immune response that is not compromised by a naturally mutating surface protein. This suggests that where new spike VOCs are not identified by neutralizing antibodies, the T-cell response may likely be cross-reactive and limit the infection (16, 25).

Other groups have reported a multivalent vaccine for SARS-CoV-2 using a viral approach coexpressing the SARS-CoV-2 Ncap and spike proteins on VSV (26) or adenovirus (27). Interestingly, the use of a multiprotein vaccine broadens the efficacy at distal organs, reducing the viral charge not only on the respiratory system but also to the distant brain (16). As stated above, nucleocapsid-specific immunity plays an indispensable role during SARS-CoV-2 infection. While antibody responses can block the initial entry of the virus at proximal sites of infection, it is the T-cell response that plays a critical role in controlling the propagation of infection, second-round infections, and subsequent viral dissemination to distal sites, providing a synergistic antiviral effect by killing virally infected cells and curtailing the further dissemination of the virus to the peripheral organs.

We are aware that our study has limitations. While an immune response was observed in two independent animal models with no toxicity and comparably strong broad immune responses, it could be argued that another model (hamster or nonhuman primate) would be needed. Access to those two models is limited, and therefore, they could not be used for the validation of our vaccine product. Additionally, we were unable to secure a facility for a challenge study, which would have strengthened our results and corroborated the effectiveness of the STX vaccine in preventing the burden associated with SARS-CoV-2 infections. A challenge study would have been important to address an antibody-dependent enhancement (ADE) effect. This is an unavoidable adverse effect of vaccines, historically observed for viruses like RSV and measles virus (28). Although ADE is a possibility, no clear data support ADE in SARS-CoV-2 infection or its occurrence in cohorts immunized against SARS-CoV-2 (29, 30). Also, due to the lack of the existence of the nucleocapsid protein on the surface of virions, it seems unlikely that the incorporation of STX-N could cause ADE, therefore suggesting a lower risk of ADE after the administration of our vaccine candidate. Finally, a direct comparison with an mRNA vaccine would have been ideal. We tried to overcome this deficit by including additional controls/samples in the neutralization assay (i.e., human plasma from infected and/or vaccinated patients); unfortunately, we were unable to use them all as desired and were limited by the quality control of the assay.

In conclusion, we rapidly generated a dual-antigen vaccine with broader immune capabilities and possible efficacy against multiple SARS-CoV-2 VOCs. The STX-S+N vaccine could eventually be used as a vaccine to boost the existing immunity generated by previously approved vaccines against spike protein while also delivering nucleocapsid immunity. Our StealthX vaccine technology, which uses rapidly engineered and manufactured exosomes to deliver nanograms of single or multiplexed viral antigens to elicit a strong, broad immune response, without any adjuvants or lipid nanoparticles, has the ability to revolutionize the next generation of vaccines.

## MATERIALS AND METHODS

### Cell lines.

Human embryonic kidney HEK 293T cells were purchased from the ATCC (ATCC CRL-3216). HEK 293T cells were maintained in culture using high-glucose Dulbecco’s modified Eagle medium (DMEM) with GlutaMAX containing 10% fetal bovine serum. HEK 293T cells were incubated at 37°C with 5% CO_2_. FreeStyle 293F cells (catalog number 51-0029; Gibco) were purchased from Thermo Fisher. 293F cells were used as a parental cell line to generate the following SARS-CoV-2 delta spike- and nucleocapsid-expressing stable cell lines: Stealth X-Spike (STX-S) and StealthX-Nucleocapsid (STX-N). Parental and engineered 293F cells were maintained in a Multitron incubator (Infors HT) at 37°C in an 80% humidified atmosphere with 8% CO_2_.

### Lentiviral vectors.

Lentiviral vectors for the expression of SARS-CoV-2 spike (delta variant B.1.617.2) (NCBI accession number OX014251.1) and SARS-CoV-2 nucleocapsid (NCBI accession number OP359729.1) were designed and synthesized by GenScript, together with two packaging plasmids (pMD2.G and psPAX2). The full-length sequences of spike and Ncap were used. Additionally, the delta spike sequence was modified for codon optimization and the insertion of a 2P mutation for the stabilization of the sequence (31). To facilitate the trafficking of spike and Ncap to the exosomes, the proteins were linked to the N terminus of the exosome-specific tetraspanin CD9 by a synthetic transmembrane domain and a secretion signal peptide; this allowed the correct membrane localization of spike and the extracellular localization of Ncap. Lentiviral particles for transduction were generated by transfecting HEK 293T cells with pMG.2 (GenScript), psPAX2 (GenScript), and STX-S_pLenti (GenScript) expressing spike or STX-N_pLenti (GenScript) expressing Ncap at a ratio of 5:5:1 using Lipofectamine 3000 according to the manufacturer’s instructions. Spike and Ncap lentiviral particles were collected at 72 h posttransfection and used to transduce 293F parental cells to generate STX-S and STX-N cells, respectively.

### Flow cytometry.

Standard flow cytometry methods were applied to measure spike and Ncap SARS-CoV-2 protein expression on the STX cell surface. In brief, 250,000 STX cells were aliquoted, pelleted, and resuspended in 100 μL eBioscience flow cytometry staining buffer (Thermo Fisher). Cells were incubated at room temperature (RT) for 30 min, with protection from light, in the presence of antispike antibody (clone 1A9, catalog number ab273433; Abcam) or antinucleocapsid antibody (catalog number ab281300; Abcam) labeled with Alexa Fluor 647 (Alexa Fluor 647 conjugation kit [fast]-Lightning-Link, catalog number ab269823; Abcam) according to the manufacturer’s protocol. Following incubation, STX cells were washed with eBioscience flow cytometry staining buffer (catalog number 00-4222-57; Thermo Fisher), resuspended in phosphate-buffered saline (PBS), and analyzed on a CytoFlex S flow cytometer (Beckman Coulter). Ten thousand events were counted per sample. Data were analyzed by using FlowJo (2021; Becton, Dickinson and Company).

### Cell sorting.

Cell sorting was performed at the Flow Cytometry Facility of the Scripps Research Institute (San Diego, CA). To enrich the spike-positive population, STX-S cells were stained as described above for flow cytometry and went through cell sorting (MoFlo Astrios EQ; Beckman Coulter) to generate pooled STX-S. The pooled STX-S was used in all of the experiments in this paper unless specified otherwise.

### STX exosome production.

STX-S and STX-N cells were cultured in FreeStyle medium (catalog number 12338018; Thermo Fisher) in a Multitron incubator (Infors HT). Subsequently, cells and cell debris were removed by centrifugation, while microvesicles and other extracellular vesicles larger than ~220 nm were removed by vacuum filtration. Next, exosomes were isolated using either Capricor’s laboratory-scale or large-scale purification method. For the laboratory-scale method, the supernatant was subjected to concentrating filtration using a Centricon Plus-70 centrifugal filter unit (catalog number UFC710008; Millipore) and then subjected to size exclusion chromatography (SEC) using a qEV original SEC column (SP5; Izon). For the large-scale method, the supernatant was subjected to concentrating tangential flow filtration (TFF) on an Äkta Flux s instrument (Cytiva, USA) and then subjected to size exclusion chromatography on an Äkta Avant 25 system (Cytiva, USA).

### Nanoparticle tracking analysis.

The exosome size distribution and concentration were determined using ZetaView nanoparticle tracking analysis (NTA) (Particle Metrix, Germany) according to the manufacturer’s instructions. Exosome samples were diluted in 0.1 μm filtered 1× PBS (catalog number 10010072; Gibco) to fall within the instrument’s optimal operating range (see Table S1 in the supplemental material).

### Jess automated Western blot analysis.

The SARS-CoV-2 spike and Ncap proteins in the cell lysate and exosomes were detected by using Protein Simple’s Jess capillary protein detection system. Samples were lysed in radioimmunoprecipitation assay (RIPA) buffer (catalog number 8990; Thermo Fisher Scientific) supplemented with a protease/phosphatase inhibitor (catalog number A32961; Thermo Fisher Scientific), quantified using the bicinchoninic acid (BCA) assay (catalog number 23227; Thermo Fisher Scientific), and run for detection. To detect spike, the separation module at 12 to 230 kDa was used according to the manufacturer’s protocol. Briefly, 0.8 μg of the sample and the protein standard were run in each capillary and probed with mouse anti-SARS-CoV-2 spike (catalog number MAB105401; R&D Systems) (1:10 dilution) or rabbit anti-SARS-CoV-2 Ncap (catalog number NBP3-00510; Novus Biologicals) (1:100 dilution), followed by secondary antibody provided in the Jess kits (horseradish peroxidase [HRP] substrate used neat).

### TEM imaging for characterization of STX exosome morphology.

STX-S and STX-N exosome samples were negatively stained on copper grids with a carbon film coating and imaged by TEM at the Electron Microscopy Core Facility at UC San Diego (San Diego, CA). Briefly, samples were treated with glow discharge, stained with 2% uranyl acetate, and dried before imaging. Grids were imaged on a JEM-1400 Plus instrument (JEOL Ltd., Japan) at 80 kV and 48 μA. Images were taken at a magnification of ×12,000 to ×80,000 with 4,000 by 4,000 pixels of resolution.

### CD81 bead assay.

STX-S or 293F parental exosomes were mixed with anti-CD81-labeled magnetic beads for 2 h at RT (catalog number 10622D; Thermo Fisher) and washed twice with PBS using a magnetic stand. Next, the bead-exosome mixtures were incubated with either directly Alexa Fluor 647-conjugated antispike antibody (see the section on flow cytometry, above), fluorescein isothiocyanate (FITC)-conjugated anti-CD81 antibody (catalog number 551108; BD Biosciences), or FITC-conjugated mouse IgGκ isotype control antibody (catalog number 555748; BD Biosciences) for 1 h at RT, followed by two PBS washes. 293F exosomes were used as a negative control for spike expression, and the isotype antibody was used as a negative control for CD81 expression. Samples were analyzed on a CytoFlex S flow cytometer (Beckman Coulter), and data were analyzed by using FlowJo (Table S2).

### Animal studies in mice.

Studies in mice were conducted in the Explora Facility (San Diego, CA) according to the guidelines of the Institutional Animal Care and Use Committee (IACUC) (protocol number EB17-004-091). To examine the efficacy of STX exosomes, age-matched BALB/c mice (female, 8 to 10 weeks old [*n* = 10 per experimental group]) were anesthetized using isoflurane and received a bilateral intramuscular (i.m.) injection (50 μL per leg, for a total of 100 μL) of either (i) PBS or (ii) STX-S, (iii) STX-N, or (iv) STX-S+N exosomes. A booster injection was performed on day 21. Mice were monitored closely for changes in health, and their weight was recorded biweekly. Blood collection was performed on day 14 and day 35. Blood (~50 to 500 μL) was collected from the submandibular vein and processed for plasma isolation after centrifugation at 4,000 rpm for 5 min at 4°C. For a comparison study, mice were injected with equal amounts of SARS-CoV-2 protein delivered as (i) soluble protein conjugated with an adjuvant (Alhydrogel at 100 μg/dose [vac-alu-250, InvivoGen]; SARS-CoV-2 Spike Protein [B.1.617.2 – delta, AntibodyOnLine]; Ncap protein, [Biolegend]) or (ii) STX exosomes. Blood was collected 2 weeks after injection and tested for IgG against SARS-CoV-2. The timeline of the mouse study is outlined in Fig. 3A. Mouse tissues (brain, salivary gland, heart, lung, liver, spleen, kidney, gastrointestinal tract [GI], and skeletal muscle [site of injection]) were collected by the Capricor Therapeutics team and fixed in 10% neutralized formalin. Tissues were sent to Reveal Biosciences for further processing and analysis. Sections were stained with hematoxylin and eosin and analyzed for alterations. Pathology evaluation was performed by Mary E. P. Goad (Fig. S2).

### Animal studies in rabbits.

The study in rabbits was conducted at CBSET, Inc., accredited by AAALAC International. All procedures and conditions of testing were performed according to USDA and animal welfare act (AWA1)/animal welfare regulation (AWR2) guidelines (IACUC protocol number I00334). To evaluate the potential toxicity and host immune response of the STX-S+N vaccine, age-matched rabbits (male and female New Zealand White, 2.5 to 3.0 kg [*n* = 8 per experimental group]) received an i.m. injection of the intended human dose (10 to 200 ng total protein in 0.5 mL) of the STX-S+N vaccine. Control animals received PBS. A booster injection was performed on day 14. Rabbits were monitored closely for changes in health, and their weight was recorded. Blood collections were performed weekly on days 0, 7, 14, 21, and 28, and blood was processed for plasma isolation after centrifugation at 4,000 rpm for 5 min at 4°C. The timeline of the rabbit study is outlined in Fig. 4A. Rabbit tissues were collected by the CBSET team at the termination of the study (day 28), fixed in 10% neutralized formalin, and analyzed for pathological alterations. A pathology report was obtained, but no images of the tissues at this stage are available; pathology scores are summarized in Table S3.

### Dose.

The doses used for immunization are reported in Table 1 (mouse) and Table 2 (rabbit); they were chosen as a consequence of a dose-response study in mice and manufacturing limitations at the time of the study. The clinical/rabbit dose of 500 μL was selected because it is 5 times the mouse dose of 100 μL; this resulted in 50 to 125 ng S and 10 to 20 ng N per dose.

### IgG ELISA.

Mouse and rabbit IgG antibodies against SARS-CoV-2 spike or Ncap were measured by enzyme-linked immunosorbent assays (ELISAs) using precoated ELISA plates (IEQ-CoV-S-RBD-IgG and IEQ-CoV-N-IgG; RayBiotech), according to the manufacturer’s instructions, at RT. Briefly, plasma samples were diluted in sample buffer (RayBiotech), added to antigen-coated wells in triplicates, and incubated at RT for 2 h on a shaker (200 rpm). Commercially available antibody against spike (catalog number S1N-S58; Acro Biosystems) or Ncap (catalog number NUN-S47; Acro Biosystems) was used as a positive control. Plates were washed 3 times with wash buffer and incubated for 1 h at RT with HRP-conjugated goat anti-mouse secondary antibodies (dilution of 1:5,000) (catalog number 115-035-003; Jackson ImmunoResearch) or anti-rabbit antibodies (dilution of 1:5,000) (catalog number 111-035-003; Jackson ImmunoResearch) diluted in assay buffer (RayBiotech). After 3 washes, the plates were developed using the TMB substrate (RayBiotech). After a 15-min incubation, the reaction was stopped by the addition of a stop solution, and the absorbance at 450 nm was recorded using a BioTek Gen5 plate reader (Agilent). Endpoint titers were calculated as the dilution that emitted an optical density (OD) exceeding 4 times that for the PBS control group.

### Neutralizing antibodies against delta SARS-CoV-2.

Samples were analyzed by Retrovirox, Inc. (San Diego, CA). Briefly, Vero E6 cells were used to evaluate the neutralization activity of the test items against a replication-competent SARS-CoV-2 delta variant (B.1.617.2). Samples were preincubated with the virus for 1 h at 37°C before addition to cells. Following the preincubation of plasma/virus samples, cells were challenged with the mixture. Samples were present in the cell culture for the duration of the infection (96 h), at which time a neutral red uptake assay was performed to determine the extent of the virus-induced cytopathic effect (CPE). Prevention of virus-induced CPE was used as a surrogate marker to determine the neutralization activity of the test items against SARS-CoV-2. Test items were evaluated in duplicates using 2-fold serial dilutions starting at a 1:40 dilution (eight total dilutions). Control wells included CoV02-delta and GS-441524, tested in singlet data points on each plate. CoV02-delta is convalescent plasma from an individual who previously received two doses of the Moderna COVID-19 vaccine before infection with the delta variant. GS-441524 is an antiviral from Gilead Sciences, which was used to validate the assay but was not included in the figures. CoV-01 is plasma from a patient who received two doses of the Moderna COVID-19 vaccine and had no history of SARS-CoV-2 infection.

### Neutralizing antibodies against omicron (BA.1 and BA.5.2.1) SARS-CoV-2.

Samples were analyzed by Retrovirox, Inc. (San Diego, CA). Neutralization assays were performed using anti-NP immunostaining (omicron BA.1 and BA.5.2.1). Briefly, samples were preincubated with the virus for 1 h at 37°C before addition to Vero E6 cells. Following incubation, the medium was removed, and the cells were then challenged with the SARS-CoV-2/test item preincubated mix. The viral inoculum was previously titrated to result in a linear response inhibited by antivirals with known activity against SARS-CoV-2. Cell culture medium with the virus inoculum was not removed after virus adsorption, and the test items and virus were maintained in the medium for the duration of the assay (48 h). Subsequently, the extent of infection was monitored by incubating cells with a human monoclonal antibody against the SARS-CoV-2 nucleocapsid protein (NP; GenScript). The amounts of the viral antigen in infected cells were estimated after incubation with horseradish peroxidase-conjugated polyclonal antibody against human IgG (HRP-conjugated goat anti-mouse IgG; BioLegend). The reaction was monitored using a colorimetric readout (absorbance at 492 nm). Test items were evaluated in duplicates using 2-fold serial dilutions starting at a 1:40 dilution. Control wells included GS-441524 (Gilead Sciences), which was tested in singlet data points on each plate to validate the assay but is not reported in the figures.

### Splenocyte isolation.

Spleens were processed for single-cell isolation by the mechanical disruption of the spleen pouch using a syringe stopper and passage through a 0.040-mm-mesh-size nylon cell strainer to remove tissue debris. Erythrocytes were lysed using ammonium chloride potassium (ACK) buffer (catalog number A1049201; Thermo Fisher), and splenocytes were collected by centrifugation. The cellular pellet was resuspended in complete RPMI 1640 medium (catalog number FG1215; Millipore Sigma-Aldrich).

### ELISpot assay.

After isolation, splenocytes were seeded at a concentration of 5E5 cells/well and incubated for 24 h in the presence or absence of 10 μg/mL of SARS-CoV-2 spike (catalog number S1N-C52H4; Acro Biosystems) or nucleocapsid (catalog number NUN-C5227; Acro Biosystems). Commercially available ELISpot plates for the evaluation of IFN-γ (for mouse: catalogue number MuIFNg [Immunospot; Cellular Technology Limited]; for rabbits: catalog number 3110-4APW-10 [MabTech]) were used. The assay was performed according to the manufacturer’s guidelines. Plates were analyzed using the S6ENTRY ELISpot reader (Immunospot; Cellular Technology Limited).

### ELISA for protein quantification.

The spike protein level on exosomes was measured by an ELISA using precoated ELISA plates (catalog number ELV-COVID19S1; RayBiotech), according to the manufacturer’s instructions, at RT. The nucleocapsid level on exosomes was measured by an ELISA using precoated ELISA plates (Legend Max SARS-CoV2 nucleocapsid protein ELISA kit, catalog number 448007; BioLegend), according to the manufacturer’s instructions, at RT. Briefly, samples and standards were loaded onto the precoated plate and incubated for 2 to 2.5 h at RT on a shaker (200 rpm). Plates were washed and incubated with a biotin-conjugated detection antibody for an hour at RT, followed by a 45-min incubation in a streptavidin solution. After washes, plates were developed using the TMB substrate. After a 30-min incubation, the reaction was stopped by the addition of a stop solution, and the absorbance at 450 nm was recorded using a BioTek Gen5 plate reader (Agilent). For nucleocapsid, after a 10-min incubation with the TMB substrate, the absorbance was recorded at 450 nm and 570 nm. For analysis, the absorbance at 570 nm can be subtracted from the absorbance at 450 nm, and the OD can be used to build a standard curve.

### Statistical analysis.

Data were analyzed using Excel and GraphPad Prism 9.1 and are shown as means ± standard errors of the means (SEM). One-way analysis of variance (ANOVA) with *post hoc* correction for multiple comparisons or a 2-tailed *t* test was applied as needed.
