# Supplementary material for: Exosome-Based Multivalent Vaccine: Achieving Potent Immunization, Broadened Reactivity, and Strong T-Cell Responses with Nanograms of Proteins
Source: Microbiol Spectr. 2023 Apr 24;11(3):e00503-23. doi: 10.1128/spectrum.00503-23 (PMC10269692; doi:10.1128/spectrum.00503-23)
Supplement: Supplemental file 1 — Fig. S1 and S2 and Tables S1 to S3. Download spectrum.00503-23-s0001.pdf, PDF file, 0.2 MB [file spectrum.00503-23-s0001.pdf]

Supplementary Figures

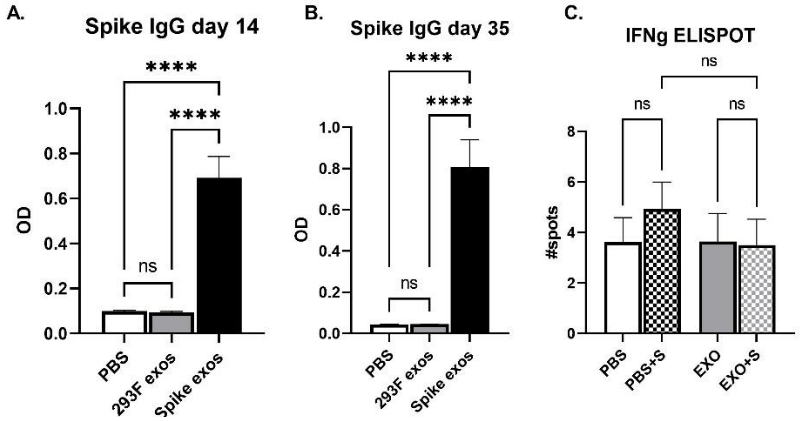

Supplementary Figure 1. Immune response to 293F exosome is comparable to PBS control. A. ELISA at day 14 after first injection. **B.** ELISA at day 35 after booster injection (2 injections) **C.** IFNγ response to PBS and exosome at day 35. Data are shown as mean ± SEM. **** p<0.001, ns= not significant, 1-way ANOVA, adjusted for multiple comparison.

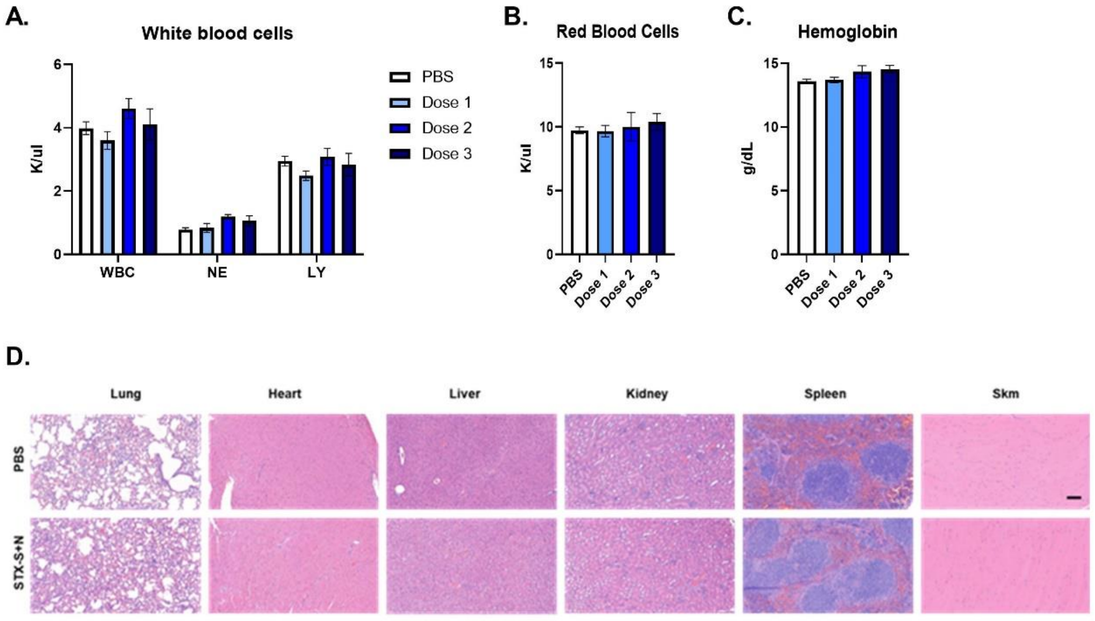

Supplementary Figure 2. No adverse effects after STX-S+N administration in mouse. A-C. Complete blood count at terminal collection. **D.** Representative images of H&E. All tissues were imaged at the same magnification. Scale bar= 100um. WBC= white blood cells; NE=neutrophils; LY= lymphocytes; RBC= red blood cells; Hb= hemoglobin; SKM= skeletal muscle. Data are shown as mean±SE. N=10 animals per experimental group.

Table S1. Analysis of exosomes by NTA.

Exosome Type	Samples	Diameter (nm)	Mean Diameter (nm)	PDI	Mean PDI
STX-S	1	140.6	144.8	0.142	0.151
	2	143.9		0.142	
	3	149.8		0.168	
STX-N	1	142.4	140.1	0.126	0.128
	2	139.3		0.137	
	3	138.5		0.122	
293F	1	132.6	133.5	0.119	0.115
	2	131.1		0.115	
	3	136.7		0.112	

Table S2. Analysis of CD81 expression on exosomes by bead-based assay.

Exosome Tested	Sample #	Average CD81 Signal (%)	Standard Deviation
STX-S	1	98.0	0.49
	2	98.5	0.42
	3	97.6	1.20
STX-N	1	99.1	0.35
	2	97.4	0.21
	3	97.4	1.13
293F	1	96.75	0.49
	2	97.8	0.85
	3	96.15	0.07

Table S3. Rabbit Pathology Summary

Treatment	Mouse	Organ Macroscopic Observation Microscopic Observation
PBS	1	No abnormal observations.
	2	No abnormal observations.
	3	No abnormal observations.
	4	No abnormal observations.
	5	No abnormal observations.
	6	No abnormal observations.
	7	No abnormal observations.
	8	No abnormal observations.
Dose 1	1	No abnormal observations.
	2	No abnormal observations.
	3	No abnormal observations.
	4	No abnormal observations.
	5	No abnormal observations.
	6	No abnormal observations.
	7	No abnormal observations.
	8	Multifocal cyst, in the limit of background alteration
Dose 2	1	No abnormal observations.
	2	No abnormal observations.
	3	No abnormal observations.
	4	No abnormal observations.
	5	No abnormal observations.
	6	No abnormal observations.
	7	No abnormal observations.
	8	No abnormal observations.
